# Telerehabilitation program for older adults on a waiting list for physical therapy after hospital discharge: study protocol for a pragmatic randomized trial protocol

**DOI:** 10.1186/s13063-021-05387-2

**Published:** 2021-07-13

**Authors:** Pollyana Ruggio Tristão Borges, Renan Alves Resende, Jane Fonseca Dias, Marisa Cotta Mancini, Rosana Ferreira Sampaio

**Affiliations:** Rehabilitation Sciences Graduate Program, Department of Physical Therapy, Universidade Federal de Minas Gerais, Avenida Antônio Carlos, 6627 - Campus Pampulha, Belo Horizonte, Minas Gerais 31270-901 Brazil

**Keywords:** Older adults, Deconditioning, Telerehabilitation, Physical therapy

## Abstract

**Background:**

Delays in starting physical therapy after hospital discharge worsen deconditioning in older adults. Intervening quickly can minimize the negative effects of deconditioning. Telerehabilitation is a strategy that increases access to rehabilitation, improves clinical outcomes, and reduces costs. This paper presents the protocol for a pragmatic clinical trial that aims to determine the effectiveness and cost-effectiveness of a multi-component intervention offered by telerehabilitation for discharged older adults awaiting physical therapy for any specific medical condition.

**Methods:**

This is a pragmatic randomized controlled clinical trial with two groups: telerehabilitation and control. Participants (n=230) will be recruited among individuals discharged from hospitals who are in the public healthcare system physical therapy waiting lists. The telerehabilitation group will receive a smartphone app with a personalized program (based on individual’s functional ability) of resistance, balance, and daily activity training exercises. The intervention will be implemented at the individuals’ homes. This group will be monitored weekly by phone and monthly through a face-to-face meeting until they start physical therapy. The control group will adhere to the public healthcare system’s usual flow and will be monitored weekly by telephone until they start physical therapy. The primary outcome will be a physical function (Timed Up and Go and 30-s Chair Stand Test). The measurements will take place in baseline, start, and discharge of outpatient physical therapy. The economic evaluations will be performed from the perspective of society and the Brazilian public healthcare system.

**Discussion:**

The study will produce evidence on the effectiveness and cost-effectiveness of multi-component telerehabilitation intervention for discharged older adult patients awaiting physical therapy, providing input that can aid the implementation of similar proposals in other patient groups.

**Trial registration:**

Brazilian Registry of Clinical Trials (ReBEC), RBR-9243v7. Registered on 24 August 2020.

**Supplementary Information:**

The online version contains supplementary material available at 10.1186/s13063-021-05387-2.

## Background

Deconditioning causes a significant functional decline in older adults and is often associated with readmissions, institutionalization, and mortality [1–3]. Deconditioning in older adults refers to a systemic physiological change after a period of inactivity and/or immobility [2, 4–6]. These changes may begin during hospitalization, as many older people are confined to bed [7, 8]. Studies demonstrate that deconditioning remains after discharge [9, 10] and the older adults who resume physical therapy immediately after leaving hospital show gains [11, 12]. The literature recommends that deconditioned older adults should receive moderate to high-intensity training focused on resistance exercises, walking, balance, and daily life activities [2].

Older people referred for rehabilitation after discharge may face long waiting lists to start treatment. The imbalance between rehabilitation supply and the growing demand for its service leads to increased waiting time for treatment [13]. This is especially common in low- and middle-income countries such as Brazil [14], whose public healthcare system serves approximately 70% of the population [15]. Specifically in the city of Belo Horizonte, located in the southeastern Brazilian state of Minas Gerais, the delay for starting physical therapy treatment in the public healthcare system can take up to 3 months. Approximately 400 individuals of different ages are waiting for physical therapy; of these, almost 70% are hospital discharges with different types of medical conditions such as postoperative of lower or upper limbs, fractures, and COVID-19. Inactivity during this waiting period may aggravate deconditioning, especially in older adults.

Telerehabilitation may be a viable alternative to reduce the waiting time for physical therapy [16], minimizing the negative impacts of inactivity, such as falls [17], sedentary pattern [18], and loss of strength, mobility, and resistance [19]. Telerehabilitation provides rehabilitation through information and communication technologies such as videoconferencing, telephoning, and smartphone app [20–22]. This rehabilitation modality offers early access to treatment, reduces costs (mostly with transportation), induces the patient to play a more active role in the treatment, and allows the treatment to be adapted to their routine [16, 23–26]. Evidence shows that telerehabilitation is safe [27, 28], effective, and less costly when compared to face-to-face rehabilitation [23, 25, 26]. In patients who underwent orthopedic surgery, telerehabilitation had positive effects on physical function [28, 29], range of motion [30], function, and pain [31]. In addition, the face-to-face and telerehabilitation modalities demonstrated equivalent results for hospital discharged patients [32–37]. More recently, the social isolation resulting from the COVID-19 pandemic has shown that distance healthcare is essential to ensure treatment continuity, especially for older adults [38]. Therefore, investing in programs that reduce costs without compromising the population’s access is essential.

This paper presents the protocol for a pragmatic superiority clinical trial that aims to determine the effectiveness and cost-effectiveness of a personalized exercise program offered by telerehabilitation to minimize the deconditioning of older adults who are, for non-specific conditions, awaiting outpatient physical therapy.

## Methods

### Study design

This is a protocol for a pragmatic randomized controlled clinical trial. Pragmatic trials are used to evaluate the effectiveness of interventions in the actual clinical practice setting. This design maximizes the application and generalization of results by establishing an appropriate basis for decision-making [39, 40]. This study protocol has been reported in accordance with the Standard Protocol Items: Recommendations for Interventional Trials (SPIRIT) Statement [41]. Additional file 1 details the SPIRIT checklist.

### Setting and participants

**Fig. 1:**
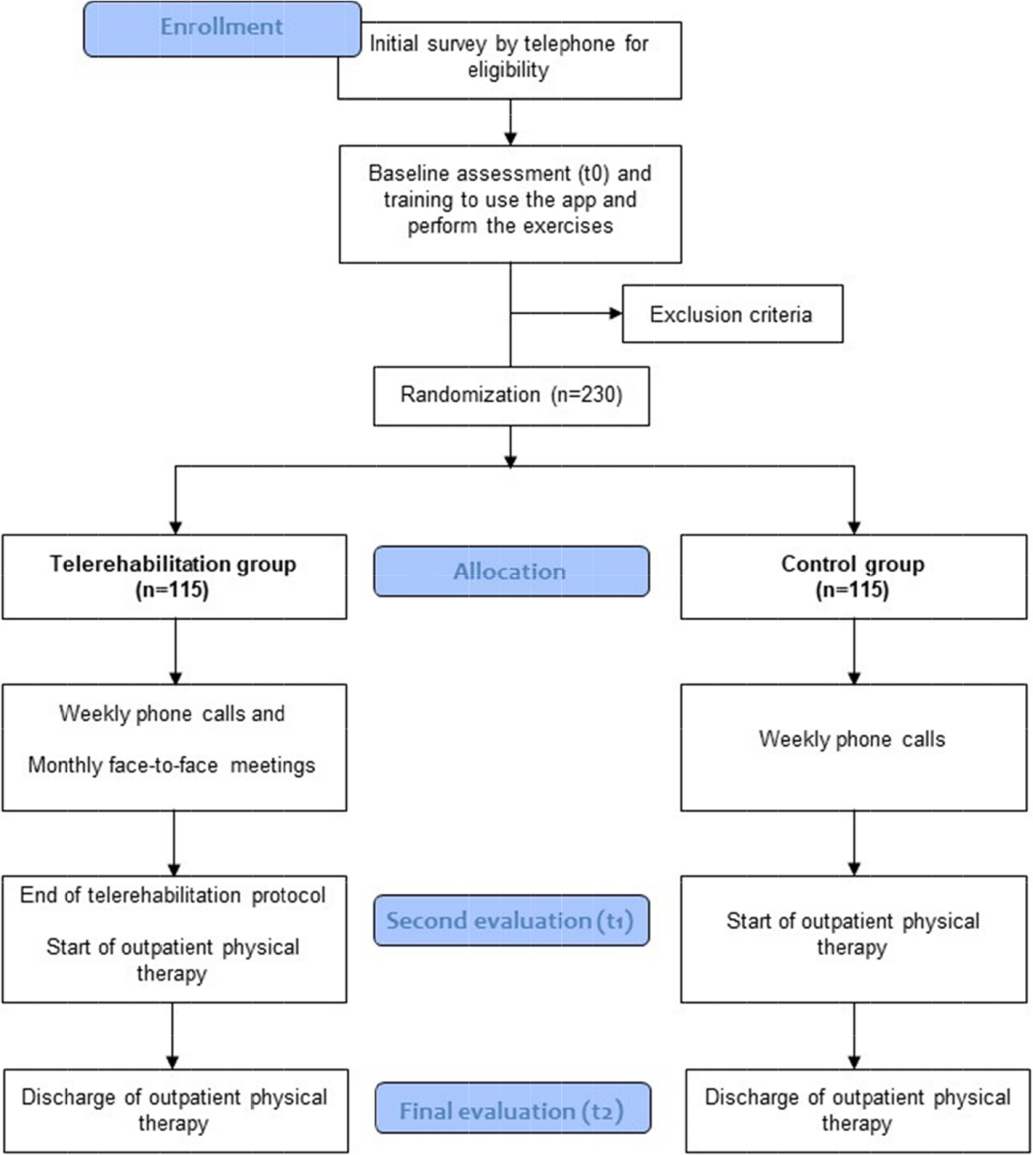
Flow diagram

Participants will be recruited from the waiting list for outpatient physical therapy of the Municipal Health Secretariat of Belo Horizonte, Brazil. All individuals on this list aged ≥ 60 years and referred to physical therapy after hospital discharge will be contacted by telephone for an initial survey to determine whether they fit the inclusion criteria with questions on mobility, cognition, and Internet access. At an in-person meeting, the researchers will detail the study and clarify any questions. The participant will then be asked to sign the consent form and will undergo the initial evaluation. Figure 1 details the study planning.

The inclusion criteria are (1) older adults age ≥ 60 years [42], (2) being on a waiting list for outpatient physical therapy in the public healthcare system for any specific medical condition, (3) have recently been discharged from the hospital, (4) no impediment to unload weight in the lower limbs to perform the telerehabilitation program, (5) walking independently or with the aid of a device and being able to sit and stand up from a chair to perform physical function tests, (6) being in a stable clinical condition to avoid complications during the telerehabilitation program, (7) having a smartphone device with Internet access (their own or a companion’s) for the telerehabilitation intervention, and (8) having a companion during the execution of the exercises at home to ensure the safety of the intervention.

The exclusion criteria are (1) the presence of clinical complications that make physical exercise impossible; 2) neurological disease, such as Parkinson or stroke; (3) a score in the Mini-Mental State Exam less than 13 for people with no schooling, 18 for individuals with low/moderate schooling, and 26 for people with a high level of schooling [43]; and (4) not being able to understand instructions or complete the tests. Excluded individuals will receive a booklet on the importance of staying active while awaiting physical therapy.

### Randomization and allocation

After the initial evaluation, participants will be randomly allocated to the following groups: telerehabilitation (TG) and control (CG). The allocation will be in random blocks sizing four and six, with an allocation rate of 1:1. The randomized sequence will be computer-generated before the start of the study by a researcher not involved in the study and placed in opaque, sealed, sequentially numbered envelopes. This sequence will remain concealed until the participant is allocated to the groups.

### Telerehabilitation intervention

The TG will receive a multi-component intervention that includes resistance exercises targeting the main muscle groups of the lower and upper limbs, and balance exercises. There are strong recommendations for the use of this type of intervention, showing improvement in clinical outcomes in older adults, such as reducing falls and increasing muscle strength and mobility [2, 17–19, 44, 45]. These exercises are easy to perform, do not require special equipment or accessories, and were designed for execution without professional supervision. In addition, the participant will choose an activity from their daily routine that they have difficulty in performing and hope to improve in the short term. The participants’ choosing an activity is a strategy aimed to stimulate engagement in the intervention and increase of their independence.

The exercise program will be carried out three to five times a week with high intensity, as suggested by Falvey et al. (2015) [2]. The volume will have three series; the number of repetitions will be customized based on Borg’s Modified Perceived Exertion Scale [46], with effort levels ranging from five to seven. The exercises’ difficulty levels and their sublevels of progression are shown in Table 1 and Additional file 2. Each participant may carry out the entire exercise program once a day or intermittently, two or three times a day (Table 1).

**Table 1:** Exercise description and progressions

EXERCISE	DESCRIPTION	PROGRESSIONS	
**Multiplanar single leg balance reach**	The participant starts this exercise in the standing position, assume single lower limb support, and move the opposite lower limb forward, to the side and backward as far as possible and maintaining the knee as straight as possible. The participant performs 3 series of 8 to 15 repetitions in each direction.	**Basic level**	1. The participant is only allowed single upper limb support on a wall or a stable surface (e.g. table or chair) for balance. The participant is allowed to touch the floor with the forefoot of the swing limb at the longest reach distance.
			2. Same as basic level 1, but with increased number of repetitions (minimum increase of 2 repetitions in each direction).
			1. Same as basic level 2, but the participant increases swing limb reach distance.
		**Moderate level**	2. Same as basic level 3, but the participant is not allowed upper limb support.
			3. Same as moderate level 1, but with increased number of repetitions (minimum increase of 2 repetitions in each direction).
			4. Same as moderate level 2, but the participant increases swing limb reach distance.
		**Advanced level**	1. Same as moderate level 3, but the participant is not allowed to touch the floor with the forefoot of the swing limb at the longest reach distance in the three directions.
			2. Same as advanced level 1, but with increased number of repetitions (minimum increase of 2 repetitions in each direction).
			3. Same as advanced level 2, but the participant increases swing limb reach distance.
**Squat**	The participant starts this exercise in the standing position with the back supported by wall and then squat. The participant performs 3 series of 8 to 15 repetitions.	**Basic level**	1. The participant perform wall squat with small range of motion (approximately 45º of knee flexion).
			2. Same as basic level 1, but with increased number of repetitions (minimum increase of 2 repetitions in each series).
			3. Same as basic level 2, but with increased range of motion (approximately 90º of knee flexion).
		**Moderate level**	1. The participant squat without back support but is allowed bilateral upper limb support on a wall or a stable surface (e.g. table or chair) for balance. In addition, the participant performs ankle plantarflexion at the end of each squat repetition (i.e. rise up on their toes).
			2. Same as moderate level 1, but with increased number of repetitions (minimum increase of 2 repetitions in each series).
			3. Same as moderate level 2, but the participant is only allowed unilateral upper limb support.
		**Advanced level**	1. The participant squat without back and upper limb support by simulating sitting on a chair with small range of motion (approximately 45º of knee flexion). In addition, the participant performs ankle plantarflexion at the end of each squat repetition (i.e. rise up on their toes).
			2. Same as advanced level 2, but with increased number of repetitions (minimum increase of 2 repetitions in each series).
			3. Same as advanced level 1, but with increased range of motion (approximately 90º of knee flexion).
**Forward lunge**	The participant starts this exercise in the standing position and then perform forward lunge alternating lower limbs. The participant performs 3 series of 8 to 15 repetitions.	**Basic level**	1. The participant performs forward lunge with small range of motion (approximately 45˚ of knee flexion) and is allowed unilateral upper limb support on a wall or a stable surface (e.g. table or chair) for balance.
			2. Same as basic level 1, but with increased number of repetitions (minimum increase of 2 repetitions in each series).
			3. Same as basic level 2, but with increased range of motion (approximately 90º of knee flexion).
		**Moderate level**	1. The participant performs forward lunge with small range of motion (approximately 45˚ of knee flexion) but is not allowed upper limb support.
			2. Same as moderate level 1, but with increased number of repetitions (minimum increase of 2 repetitions in each series).
			3. Same as moderate level 2, but with increased range of motion (approximately 90º of knee flexion).
		**Advanced level**	1. The participant performs forward lunge with small range of motion (approximately 45˚ of knee flexion) and simultaneous trunk rotation to the side of the limb that moves forward. Upper limb support is not allowed.
			2. Same as advanced level 1, but with increased number of repetitions (minimum increase of 2 repetitions in each series).
			3. Same as advanced level 2, but with increased range of motion (approximately 90º of knee flexion).
**Lateral lunge**	The participant starts this exercise in the standing position and then perform lateral lunge alternating lower limbs. The participant performs 3 series of 8 to 15 repetitions.	**Basic level**	1. The participant performs lateral lunge with small range of motion (approximately 30˚ of knee flexion) and is allowed bilateral upper limb support on a wall or a stable surface (e.g. table or chair) for balance.
			2. Same as basic level 1, but with increased number of repetitions (minimum increase of 2 repetitions in each series).
			3. Same as basic level 2, but with increased range of motion (approximately 60º of knee flexion).
		**Moderate level**	1. The participant performs lateral lunge with small range of motion (approximately 30˚ of knee flexion) but is not allowed to use upper limb support for balance.
			2. Same as moderate level 1, but with increased number of repetitions (minimum increase of 2 repetitions in each series).
			3. Same as moderate level 2, but with increased range of motion (approximately 60º of knee flexion).
		**Advanced level**	1. The participant performs lateral lunge with small range of motion (approximately 30˚ of knee flexion) and simultaneous trunk rotation to the side of the limb that moves to the side. Upper limb support is not allowed.
			2. Same as advanced level 1, but with increased number of repetitions (minimum increase of 2 repetitions in each series).
			3. Same as advanced level 2, but with increased range of motion (approximately 60º of knee flexion).
**Wall/kneeling/floor pushup**	The participant starts this exercise in the standing position with feet shoulder-width apart and facing a wall free from any objects or obstacles. The participant is just over one arm’s lengths away from the wall (30 to 45 centimeters). Then, the participant put the palms of both hands flat against the wall at shoulder height, approximately shoulder-width apart, and then lower and lift him/herself against the wall while keeping their feet planted firmly on the floor and maintaining the back and hips straight. The progression to advanced level includes knelling and floor pushup. The participant performs 3 series of 8 to 15 repetitions.	**Basic level**	1. The participant performs bilateral wall pushup with small range of motion (approximately 45˚ of elbow flexion).
			2. Same as basic level 1, but with increased number of repetitions (minimum increase of 2 repetitions in each series).
			3. Same as basic level 2, but with increased range of motion (approximately 90º of elbow flexion).
		**Moderate level**	1. The participant performs unilateral wall pushup with small range of motion (approximately 45˚ of elbow flexion).
			2. Same as moderate level 1, but with increased number of repetitions (minimum increase of 2 repetitions in each series).
			3. Same as moderate level 2, but with increased range of motion (approximately 90º of elbow flexion).
		**Advanced level**	1. The participant performs kneeling pushups with approximately 90˚ of elbow flexion.
			2. Same as advanced level 1, but with increased number of repetitions (minimum increase of 2 repetitions in each series).
			3. The participant performs floor pushups with approximately 90˚ of elbow flexion.


**Additional file 2.** Exercise protocol video.

The exercises will be available in a smartphone app developed for this study. This technology is widely used, being the main form of Internet access [47]. The app will be installed on either the participant’s or the companion’s smartphone. After each exercise, the participants will report their level of pain and difficulty on the app, on a scale of 0 to 5. The exercise program may also be accessed via a computer if the participant prefers this device. After the initial evaluation, participants and their companions will be trained in how to use the app and perform the exercises. Information on deconditioning and the benefits of the proposed exercises will also be provided.

Two previously trained physical therapists who have a master’s degree will be responsible for monitoring the intervention. The proposed exercises and activities will be performed at home, in the presence of a companion to increase the safety of the exercises and facilitate the management of the app. Participants will be monitored by telephone and in-person meetings. The phone calls will take place weekly to monitor the exercise execution, pain, and adverse events. The progression of the exercises’ difficulty through the protocol’s sublevels will be based on this information, as well as those reported daily in the app. The face-to-face meetings will occur monthly (between 24 and 30 days) at the participant’s rehabilitation center. In these meetings, the professionals will evaluate the execution of the exercises and adjust the difficulty level according to the participant’s performance. The intervention will cease when the participant is contacted to begin outpatient physical therapy. Therefore, the participation period in the trial will depend on the waiting time of each participant.

### Control group

CG participants will follow the usual flow of the waiting list of patients awaiting outpatient physical therapy in the public healthcare system and will not receive any guidance or exercise, as recommended in pragmatic studies. They will be monitored weekly by telephone call to check the onset of outpatient physical therapy and adverse events. Like the TG, the period each CG participant will spend in the trial will depend on the time awaiting outpatient physical therapy. Participants in both the TG and CG will not be prohibited from receiving others interventions such as exercises on their own, physical activity (e.g., walking), medications, or physical therapy elsewhere while on the waiting list, but this information will be recorded.

### Data collection

**Fig. 2:**
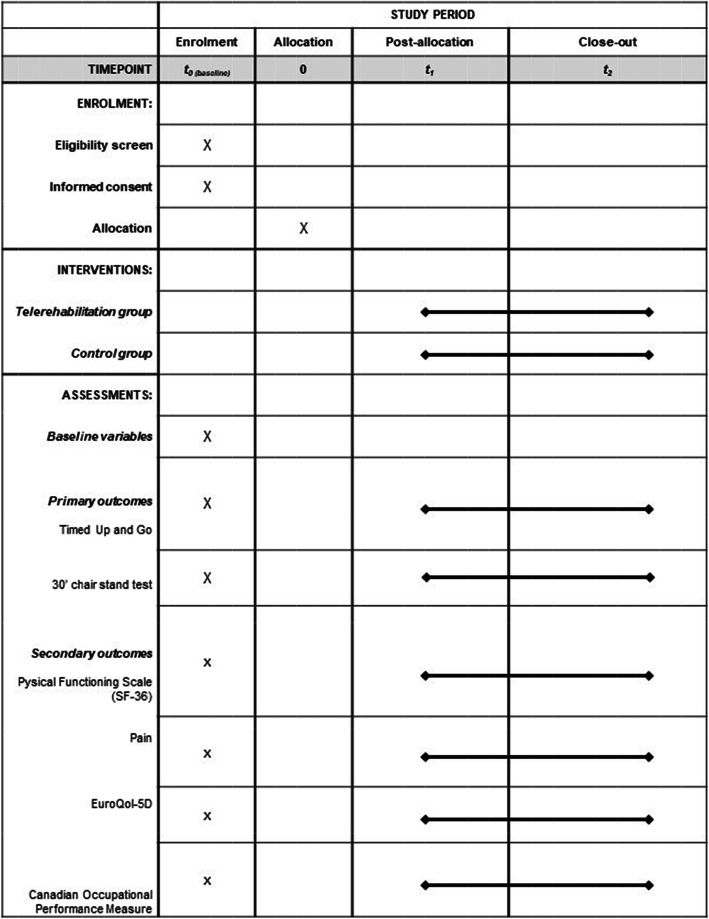
Schedule of enrolment, interventions, and assessments (according to SPIRIT)

Figure 2 shows the schedule of enrollment, interventions, and assessments (according to the SPIRIT). Outcomes will be assessed by in-person meetings at baseline (t0), at the end of the telerehabillitation protocol (t1), and after discharge from outpatient physical therapy or within 6 months of outpatient treatment (t2). All evaluations will be performed by a trained evaluator blinded to group allocation. All instruments show adequate validity and reliability for older adults [48–52].

In the baseline (t0), besides the outcomes, socio-demographic and clinical data will be collected, as well as data on the companion, and information on the use of communication technologies.

In the second evaluation (t1), satisfaction with the intervention and the TG app will also be evaluated. To minimize sample loss, the second evaluation will occur before the start of outpatient physical therapy or up to 1 week after the start of physical therapy treatment. The start of the outpatient physical therapy will be accompanied by weekly phone calls to both groups.

The final evaluation (t2) will take place on the last day of outpatient physical therapy or up to 1 week after discharge. The evaluation will consist of the collection of outcomes and information about outpatient physical therapy. Reasons for program discontinuity and follow-up losses will be recorded throughout the study.

### Outcomes

#### Primary outcome

The primary outcome will be physical function measured by the Timed Up and Go (TUG) and the 30-s Chair Stand Test (30CST). These tests were chosen because they are simple, fast, widely used and do not require special equipment.

The TUG was developed to measure the functional mobility of older people [53]. The participant gets up from a chair and walks 3 m alone or with the aid of a walking device, at a comfortable and safe speed, and then return and sit down again in the same chair [53].

The 30CST consists of sitting and standing, tasks often performed daily [54, 55]. This test was developed as a measure of lower limb strength for older adults [49]. The participant sits in a chair with arms crossed in front of the torso and must repeatedly stand and sit for 30 s as fast as possible [56].

#### Secondary outcomes

The Physical Functioning Scale of the Brazilian version of the Medical Outcomes Short-Form Health Survey (SF-36) will be used to measure physical health [57]. The Physical Functioning Scale presents ten questions; each one is scored from 1 to 3 according to the perceived limitation [58]. The final score of the scale is calculated according to the orientation of the SF-36 authors ranges from 0 to 100, with lower scores indicating higher limitations [58].

Pain will be measured by the visual analog scale with numerical (0–10) and color gradations [59]. Health-related quality of life will be measured by the Brazilian version of EuroQol-5D (EQ-5D-3L). This instrument evaluates the patient’s current health status in five dimensions (mobility, self-care, usual activity, pain/discomfort, and anxiety/depression). EQ-5D-3L also includes a visual analog scale to record overall health status [60]. The results of this instrument are widely used in economic evaluations [61].

Performance perception and satisfaction will be measured by the Canadian Occupational Performance Measure (COPM) [62]. In this study, only the self-care area will be used. The participant lists the self-care activities they have difficulty performing, chooses the one they consider the most important, and ranks their performance and performance satisfaction from 0 to 10. Higher scores indicate better performance and performance satisfaction.

### Economic evaluation

The economic evaluation will be carried out from the perspective of society and the Brazilian public healthcare system during the study period. The costs will be reported based on values updated to the year of data collection. In this phase, all information will follow the recommendations of the Brazilian Ministry of Health’s Methodological Guidelines for Economic Evaluation [63]. Cost measures will be obtained by estimates of health service utilization (public and private) due to the health condition that caused the referral to outpatient physical therapy and/or generated adverse events. The app development costs will be excluded.

The costs to the public healthcare system will be measured based on the use of health services and procedures reported by participants by the weekly Google form platform, and these data will only be assessed by researchers blinded to group allocation. The costs for hospital care, elective consultations, outpatient or home physical therapy, and medication will be included. These costs will be valued using standardized cost tables of the Brazilian public healthcare system.

Costs to society will include direct costs to participants and caregivers and loss of productivity. The calculation of direct expenses, such as medication, purchase of equipment, transportation, hiring caregivers, and health services will be based on surveys answered by participants. The cost of transportation will be evaluated by the distance between the participant’s home and the physical therapy site, adjusted by the type of transportation used and the price of gasoline. Information on the loss of productivity will be obtained for participants and non-contracted caregivers (e.g., family members) through surveys administered to participants who will indicate whether they have paid work, income from this work, and lost workdays due to the health condition.

The cost-consequence approach will be used to compare the results obtained by the participants in the primary and secondary outcomes with the costs for the two perspectives of analysis (society and public healthcare system). In the cost-utility analysis, the costs for the public healthcare system will be compared with the quality-adjusted life years (QALY) gained from the intervention, obtained by applying the EQ-5D-3L. Sensitivity analyses will be conducted to explore any degree of uncertainty in the estimates, such as sampling, time horizon, discount rate, and imputation of missing data.

### Other outcomes

Adherence will be monitored through the records computed by the app and confirmed during the weekly calls to the TG. Participants who perform all the proposed exercises at least three times a week will be considered fully adherent.

Adverse events will be monitored weekly during phone calls and will be reported in the study results. Adverse events are those harmful or unfavorable results that occur during or after exercise [64], such as nausea, headache, falling, and incapacitating pain. Events not related to the intervention will also be monitored. Participants who report a serious adverse event will be instructed to seek emergency medical services. The intervention will be interrupted if the adverse event makes it impossible for the participant to perform physical exercises.

### Sample size

The sample size was based on the TUG primary outcome. An effect size of d=0.4, previously obtained by Brovold et al. (2012) [65], power of 80%, alpha of 0.05, and abandonment rate of 15% were considered to estimate sample size using the G*Power 3.1 software. This resulted in a minimum sample size of 230 participants, 115 per group.

### Data analysis plan

The outcome variables and the participants’ characteristics will be analyzed with descriptive statistics. The Kolmogorov-Smirnov test will be used to test the normality of the data. Comparison of TG and CG baselines will be conducted with parametric or non-parametric test.

Outcome group comparisons will be analyzed with the nonlinear mixed effect model [66], given that deconditioning follows a nonlinear path over time and the period individuals remain in the study is not homogeneous. Since the intervention time will be different among the participants, time will be treated as a random factor.

All data will be analyzed for intention-to-treat; that is, all random participants will be included in the analysis regardless of adherence to the protocol. The data will be analyzed by the R software (R Foundation for Statistical Computing, Vienna, Austria) with a significance level of 5%.

## Discussion

### Potential impact and significance

Our hypothesis is that the multi-component program offered by telerehabilitation will minimize the deconditioning of hospital discharged older adult patients waiting for physical therapy and will reduce costs. Older adults who were hospitalized due to different health conditions and who experienced functional decline have up to five times greater likelihood of dying and 10 to 20 times greater chance of being readmitted after hospital discharge [67–69]. In addition, studies show that approximately 30% of these older individuals have functional losses at the moment of hospital discharge [68, 69] and that roughly the same proportion maintain these losses 30 days after discharge [69]. The type of intervention most recommended for this population is a multicomponent program [2, 45]. Thus, the faster these older individuals start physical therapy and remove themselves from inactivity, the less negative will be the impacts and deconditioning advancement. The costs for patients and the public healthcare system may be reduced. Telerehabilitation reduces the cost of transportation [23–25]. Once deconditioning is associated with readmissions [1–3] and hospitalization generates cost to the healthcare system, the exercise program may reduce hospital readmissions.

In Brazil, telerehabilitation is still not a common practice, with a higher concentration of investments aimed towards telehealth. In low- and middle-income countries, studies using the remote rehabilitation are incipient. Due to the COVID-19 pandemic, this rehabilitation modality has been gaining prominence as an alternative to face-to-face treatment [70]. The pandemic has impacted health services around the world, particularly non-urgent treatments. There is no estimate regarding the end of social isolation measures in many countries, and it is not possible to predict when healthcare services will return to normality. Waiting lists for outpatient services will possibly be even longer after the pandemic. As such, telerehabilitation is becoming increasingly essential to ensure treatment for the general population.

### Strengths and weakness

This pragmatic study will be developed in the context of the public healthcare system, which favors—if our hypothesis is proven right—a simple and direct application in clinical practice. To reduce the risk of bias, the protocol will be prospectively recorded, the sample size will be representative of the population of interest, participants will be randomly allocated to groups, the allocation process will be concealed, the evaluators will be blinded to the group allocation, and data will be analyzed by intention-to-treat. Blinding participants and physical therapists to the intervention will not be possible due to the nature of the intervention.

### Contribution to physical therapy

We expect the clinical trial will provide evidence on the effectiveness and cost-effectiveness of a multi-component telerehabilitation program for hospital discharged older adult patients awaiting outpatient physical therapy in the Brazilian public healthcare system. In addition, the study is expected to trigger discussions and the implementation of similar interventions in different patient groups and locations.

### Trial status

Protocol version 1, date: August 24, 2020. Recruitment will start on February 22, 2021, http://www.ensaiosclinicos.gov.br/rg/RBR-9243v7. Recruitment completion is expected by December, 2021. The results of this trial will be submitted to a peer-reviewed journal after sample size is complete.

## Supplementary Information

**Additional file 1.** SPIRIT Checklist.

## Data Availability

The datasets used and/or analyzed during the current study will be available from the corresponding author on reasonable request.

## Abbreviations

30CST: 30-Second Chair Stand Test
CG: Control group
COPM: Canadian Occupational Performance Measure
EQ-5D-3L: EuroQol-5D
QALY: Quality-adjusted life years
SF-36: Medical Outcomes Short-Form Health Survey
SPIRIT: Standard Protocol Items: Recommendations for Interventional Trials
TG: Telerehabilitation group
TUG: Timed Up and Go
